# Supplementary material for: Objectively Measured Physical Activity among People with and without HIV in Uganda: Associations with Cardiovascular Risk and Coronary Artery Disease
Source: Glob Heart. 2025 Feb 10;20(1):13. doi: 10.5334/gh.1398 (PMC11827560; doi:10.5334/gh.1398)
Supplement: Supplementary Content. — Supplementary Figures 1 to 3. [file gh-20-1-1398-s1.pdf]

Supplementary Figure 1. Depicts flow of data analysis among study participants. Objective physical activity metrics were used for analysis. Abbreviations: ASCVD, atherosclerotic cardiovascular disease risk; CAD, coronary artery disease.

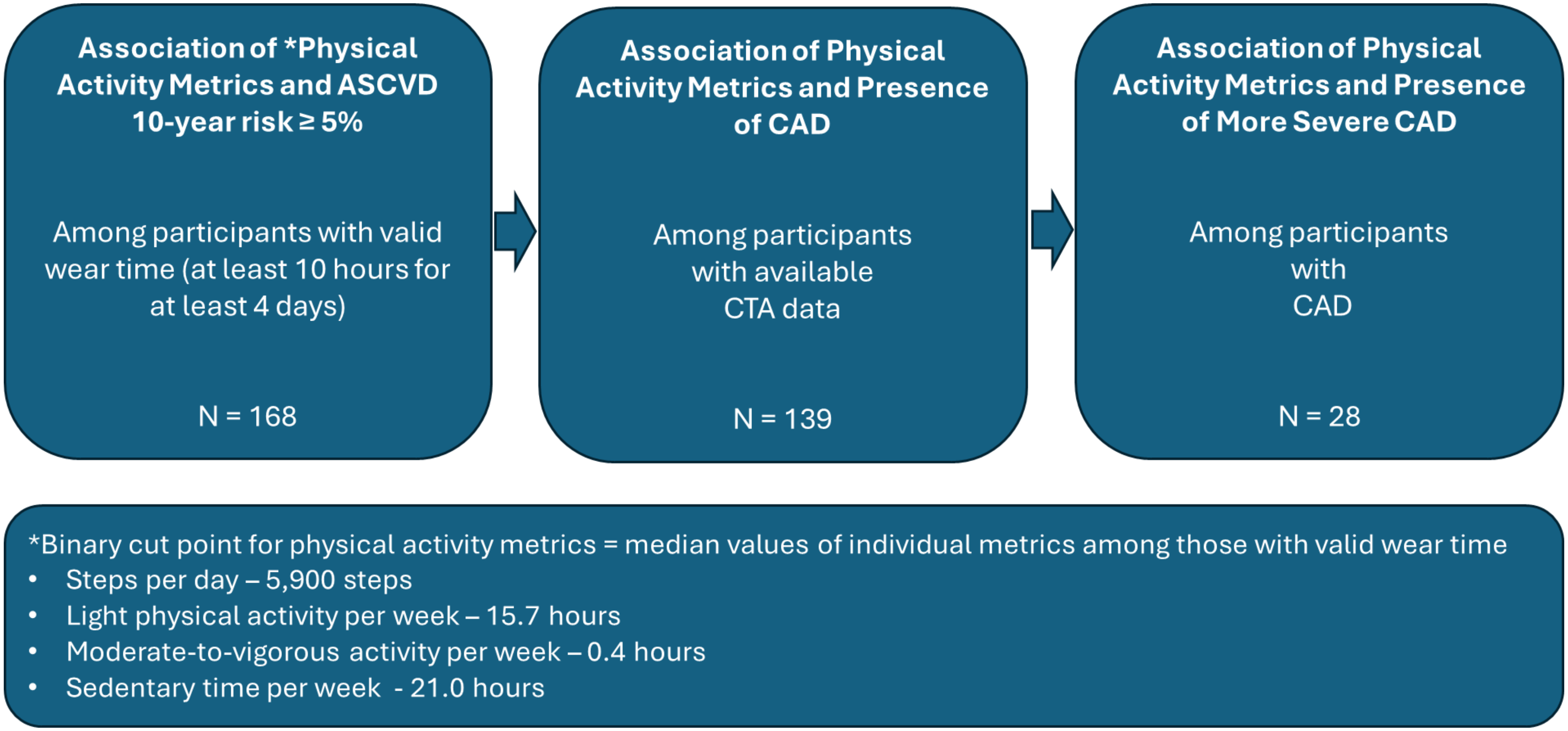

Supplementary Figure 2. Relationship between self-reported and objective measures of physical activity. (a), (b), and (c) demonstrate scatter plots and Spearman's correlation for self-reported vs objectively measured sedentary, light activity, and moderate-to-vigorous activity time, respectively. (d), (e), and (f) demonstrate Bland-Altman plots discerning the degree of agreement between self-reported vs objectively measured sedentary, light activity, and moderate-to-vigorous activity time.

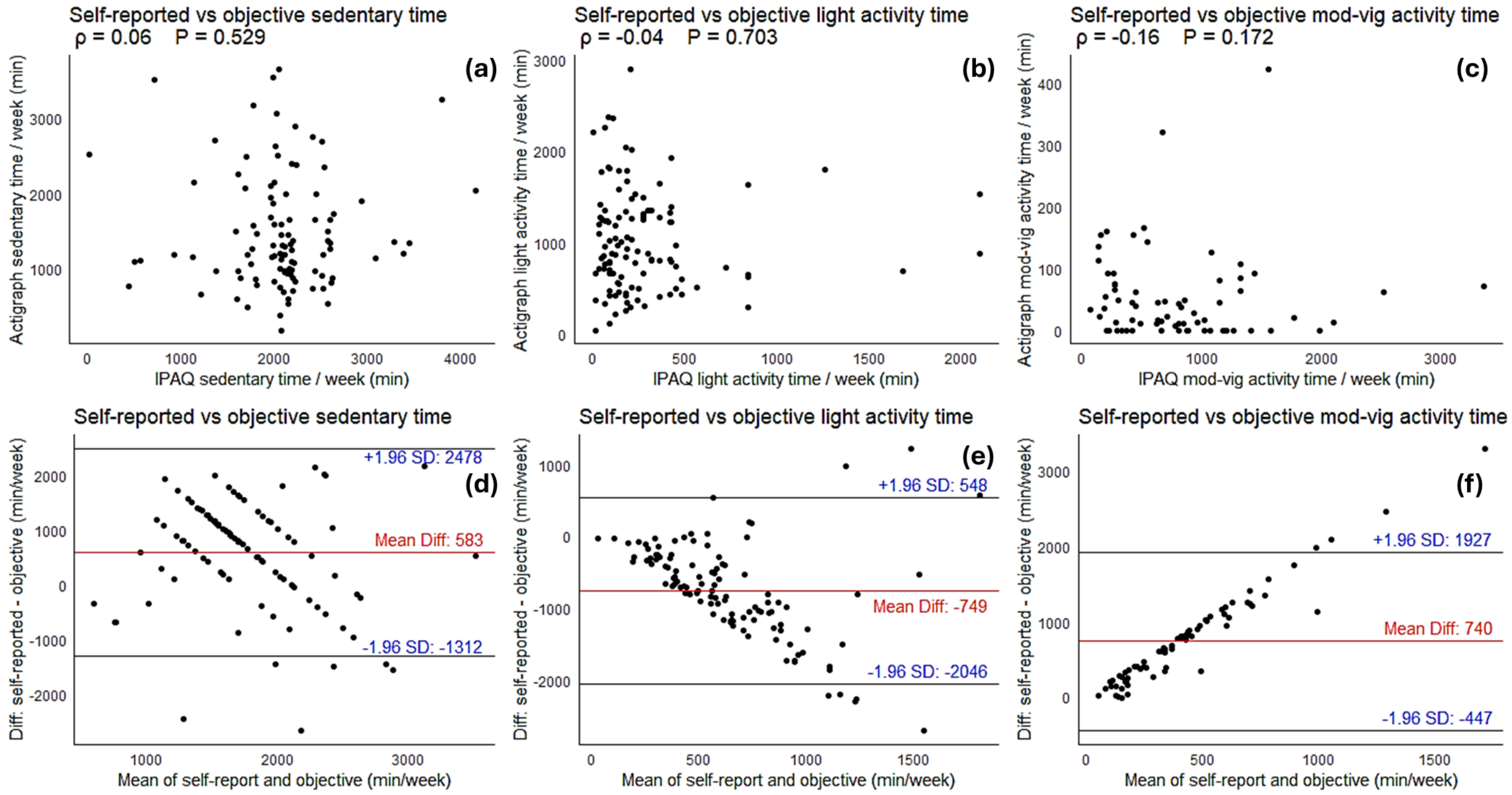

Definitions: ρ = rho = Spearman's coefficient; Diff, difference; SD, standard deviation

Supplementary Figure 3. Correlation of age with objective measures of (a) sedentary time, (b) light activity time, (c) moderate-to-vigorous activity time, and (d) steps per day.

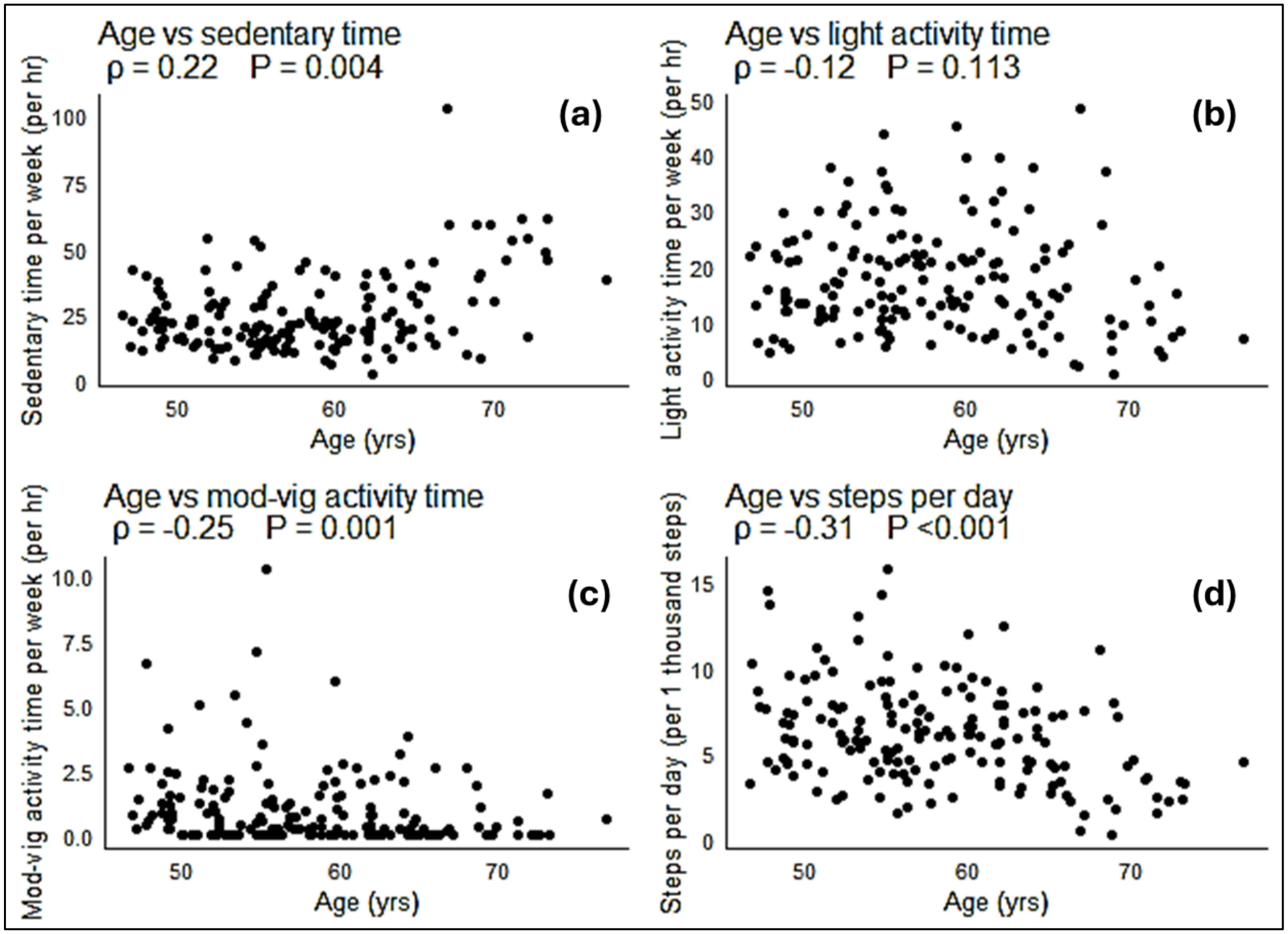

Definitions: ρ = rho = Spearman's coefficient
